# Huntington's disease clinical trials update: October 2025

**DOI:** 10.1177/18796397251399751

**Published:** 2025-11-26

**Authors:** Mena Farag, Sarah J Tabrizi, Edward J Wild

**Affiliations:** 1 Huntington's Disease Centre, 61554 UCL Queen Square Institute of Neurology, London, UK

**Keywords:** Huntington's disease, clinical trials

## Abstract

In this edition of the Huntington's Disease Clinical Trials Update, we expand on the launch of the phase II/III clinical trial of SKY-0515 from Skyhawk Therapeutics and the phase I/II clinical trial of SPK-10001 from Spark Therapeutics. We also report positive topline data from uniQure's phase I/II clinical trial of AMT-130 after 36 months of follow-up. Further updates include recent developments in Roche's tominersen programme within GENERATION HD2, progress with votoplam (PTC518) in PIVOT-HD by PTC Therapeutics and developments in the collaborative PTC Therapeutics/Novartis programme. We additionally discuss regulatory developments regarding pridopidine following the negative PROOF-HD study. Finally, we provide an updated listing of all registered and ongoing clinical trials in Huntington's disease.

## Introduction

The Clinical Trials Update is a regular feature devoted to highlighting ongoing and recently completed clinical trials in Huntington's disease (HD). Clinical trials previously reviewed in this section are listed in Table 1. For current and completed studies discussed here, endorsement status by the European Huntington's Disease Network (EHDN) is now also indicated.

**Table 1.** Clinical trials previously reviewed by the Huntington's disease clinical trials update.

	Trial Name	Intervention	Endorsed by EHDN	Edition
NCT02519036	IONIS-HTTRx	IONIS-HTT_Rx_^a^	Yes	September 2017^1^
NCT02215616	LEGATO-HD	Laquinimod	Yes	
NCT02197130	Amaryllis	PF-02545920	Yes	
NCT02006472	PRIDE-HD	Pridopidine	Yes	
NCT03225833	PRECISION-HD1	WVE-120101	Yes	February 2018^2^
NCT03225846	PRECISION-HD2	WVE-120102	Yes	
NCT01795859	FIRST-HD	Deutetrabenazine	No	
NCT02481674	SIGNAL	VX15/2503	No	August 2018^3^
NCT00712426	CREST-E	Creatine	No	
NCT03761849	GENERATION HD1	RG6042^a^	Yes	January 2019 ^4^
NCT03344601	PACE-HD	Physical activity	Yes	
NCT02535884	HD-DBS	Deep brain stimulation	Yes	June 2019^5^
NCT02453061	TRIHEP3	Triheptanoin	No	
NCT04120493	AMT-130	AAV5-miHTT	Yes	April 2020^6^
NCT04102579	KINECT-HD	Valbenazine	No	
NCT05111249	VIBRANT-HD	Branaplam	Yes	April 2022^7^
NCT04514367	ANX005	ANX-005	No	
NCT04406636	SHIELD HD	Observational study	Yes	
NCT03761849	GENERATION HD1	Tominersen^a^	Yes	
NCT05032196	SELECT-HD	WVE-003	Yes	
NCT03225833	PRECISION-HD1	WVE-120101	Yes	
NCT03225846	PRECISION-HD2	WVE-120102	Yes	
NCT02481674	SIGNAL	Pepinemab^b^	No	November 2022^8^
NCT05358717	PIVOT-HD	PTC518	Yes	
NCT05686551	GENERATION HD2	Tominersen^a^	Yes	August 2023^9^
NCT05541627	AB-1001	AAVrh10.CAG.hCYP46A1^c^	No	
NCT05822908	VO659-CT01	VO659	No	March 2024^10^
NCT05111249	VIBRANT-HD	Branaplam	Yes	
NCT06254482	PIVOT-HD	PTC518 (extension study)	Yes	September 2024^11^
NCT06585449	ALN-HTT02	ALN-HTT02	Yes	March 2025^12^
NCT05107128	DIMENSION	SAGE-718	Yes	
NCT05655520	PURVIEW	SAGE-718	Yes	
*NCT06873334	FALCON-HD	SKY-0515	No	October 2025
*NCT06826612	N/A	SPK-10001	No	

^a^ IONIS-HTT_Rx_, RG6042 and tominersen refer to the same molecule.

^b^ VX15/2503 and pepinemab refer to the same molecule.

^c^ AAVrh10.CAG.hCYP46A1, BV-101 and AB-1001 refer to the same molecule.

In this edition, we expand on the launch of the phase II/III clinical trial of SKY-0515 (NCT06873334)^13^ (FALCON-HD) from Skyhawk Therapeutics and the phase I/II clinical trial of SPK-10001 (NCT06826612)^14^ from Spark Therapeutics. We also report positive topline data from uniQure's phase I/II clinical trial of AMT-130 (NCT04120493; NCT05243017)^15,16^ after 36 months of follow-up. Further updates include recent developments in Roche's tominersen programme within GENERATION HD2 (NCT05686551),^17^ progress with votoplam (PTC518) in PIVOT-HD (NCT05358717; NCT06254482)^18,19^ by PTC Therapeutics and developments in the collaborative PTC Therapeutics/Novartis programme. We additionally discuss regulatory developments regarding pridopidine following the negative PROOF-HD (NCT04556656)^20^ study. Finally, we provide an updated listing of all registered and ongoing clinical trials in Huntington's disease.

We tabulate all currently registered and ongoing clinical trials in Tables 2–4. For further details on the methodology used, please refer to the first edition of this series.^1^

**Table 2.** Pharmacological clinical trials registered at the World Health Organization (WHO) International Clinical Trials Registry Platform (ICTRP) for people with Huntington's disease (HD) since the first edition of the “Huntington's Disease Clinical Trials Corner”.

Registration ID	Trial Name	Intervention	Mechanism of Action	Population	Comparison	Main Outcome	Study Design	Estimated Enrolment	Sponsor	Location(s)
NCT06873334*	FALCON-HD	SKY-0515	mRNA splicing modulator to lower mHTT and PMS1	Early HD; UHDRS TFC ≥10	Placebo	Changes in blood mHTT, brain volume (MRI) and UHDRS at 12 months	Randomized, double-blind, placebo-controlled, dose-ranging, parallel assignment	120	Skyhawk Therapeutics, Inc.	Australia, New Zealand (multi-centre)
NCT06853743*	NAD-HD	Nicotinamide riboside	Vitamin B3 derivative; NAD^+^ precursor	Early and moderate HD	Placebo	Change in composite UHDRS at 24 months	Randomized, double-blind, placebo-controlled, parallel assignment	120	Oslo University Hospital	Norway (single centre)
NCT06826612*	-	Intraparenchymal infusion of SPK-10001 into caudate and putamen bilaterally	AAV-mediated gene therapy delivering therapeutic construct to striatum	HD-ISS stage 1 or 2 HD; UHDRS TFC ≥11	Placebo/sham surgery	Change in UHDRS TFC; safety/TEAEs	Randomized, sequential dose-escalation, quadruple-masked, parallel assignment	53	Spark Therapeutics, Inc.	USA (multi-centre)
NCT06585449	-	ALN-HTT02	RNA interference selectively targeting exon 1 of *HTT* mRNA	HD-ISS stage 2 or early stage 3 HD	Placebo	Frequency of TEAEs in both the double-blind and open-label phases, monitored over 12 months	Randomized, double-blind, parallel assignment, single dose	54	Alnylam Pharmaceuticals	Canada, Germany, UK (multi-centre)
CTIS2024-514328-18-00	VO659-CT01	Intrathecal administration of VO659	ASO targeting mutant *HTT* and ataxin RNA to reduce toxic protein levels	SCA1, SCA3, HD	None	Safety, tolerability, and pharmacokinetics of multiple doses	Open-label, non-randomized	23	Vico Therapeutics B.V.	Denmark, France, Germany, Israel, Netherlands, UK (multi-centre)
CTIS2024-518875-73-00	TEMET-HD	Metformin	Modulation of cellular energy metabolism via AMP-activated protein kinase activation	HD	Placebo	Change in UHDRS cognitive score after 6 and 12 months	Randomized, double-blind, placebo-controlled	60	IIS La Fe	Spain (pending)
NCT06474650	—	LPM3770164	VMAT2 inhibitor	Healthy controls	None	Pharmacokinetics pre-dose and up to 240 h post-dose	Randomized, open-label, two-period, double-crossover (phase I) study	16	Luye Pharma Group Ltd	China (single centre)
NCT06469853	—	MBF-015	Histone deacetylase 1/2 inhibitor	Early and moderate HD	None	Safety and tolerability at 43 days	Open-label, single centre (phase IIa) study	10	Medibiofarma S.L.	Spain (single centre)
NCT06312189	—	Valbenazine	VMAT2 inhibitor	HD with chorea; participated in study NBI-98854-HD3006 (NCT04400331)	None	Number of participants with TEAEs up to week 106	Non-randomized, open-label	7	Neurocrine Biosciences	Canada (multi-centre)
NCT06254482	—	PTC518	Small molecule splicing modulator	Participants who completed the treatment period in PTC518-CNS-002-HD	None	Number of participants with TEAEs up to month 30; blood total HTT levels up to month 28	Randomized, double-blind, parallel assignment, extension (phase IIb) study	250	PTC Therapeutics	Australia, Austria, Canada, France, Germany, Italy, Netherlands, New Zealand, Spain, UK (multi-centre)
NCT06097780	—	Nestacell	Dental pulp stem cell	Early and moderate HD	Placebo	Efficacy at 1 year	Randomized, double-blind, parallel assignment, multiple dose	120	Azidus Brasil	N/S
NCT06024265	—	ER2001	Small interfering RNA	Early HD	None	Safety at 6.5 months	Multiple dose, open-label trial	15	ExoRNA Bioscience	China (single centre)
2022-001565-12	—	PTC518	Small molecule splicing modulator	Premanifest, prodromal and early HD	None	Safety at 24 months, blood total HTT levels at 24 months	Randomized, double-blind, parallel assignment, multiple dose	250	PTC Therapeutics	France, Germany, Netherlands, UK, USA (multi-centre)
NCT05822908	—	VO659	CAG-targeting ASO	Early HD, mild-moderate SCA1, mild-moderate SCA3	None	Safety at 253 days	Open-label, non-randomized, sequential assignment, multiple ascending dose	65 (19 HD, 19 SCA1 and 27 SCA3)	VICO Therapeutics B.V.	France, Germany, Italy, Poland, the Netherlands, UK (multi-centre)
NCT04556656**†**	PROOF-HD	Pridopidine	Sigma-1 receptor activation	Early HD	Placebo	Change in function at 65 weeks	Randomized, double-blind, parallel assignment, single dose trial	499	Prilenia Therapeutics	Austria, Canada, Czechia, France, Germany, Italy, Netherlands, Poland, Spain, UK, USA (multi-centre)
NCT05686551	GENERATION HD2	Tominersen	Non-allele-selective ASO	Prodromal and early HD	Placebo	Safety at 24 months	Randomized, double-blind, dose-finding trial	360	Hoffmann-La Roche	USA, Spain, more sites to be confirmed (multi-centre)
NCT05655520**†**	PURVIEW	SAGE-718	Positive allosteric modulator of NMDA	Premanifest, early and moderate HD	None	Safety at 13 months	Single-dose open-label trial	153	Sage Therapeutics	Canada, USA (multi-centre)
NCT03019289**†**	—	Pridopidine	Sigma-1 receptor activation	Healthy controls, early and moderate HD	None	Sigma-1 receptor occupancy	Multiple dose, open-label trial	23	Prilenia Therapeutics / Teva	Germany (single centre)
NCT02494778**†**	Open PRIDE HD	Pridopidine	Sigma-1 receptor activation	Early and moderate HD	Placebo	Efficacy at 106 weeks	Open-label extension	400	Prilenia Therapeutics / Teva	Australia, Austria, Canada, France, Germany, Italy, Netherlands, Poland, Russia, UK, USA (multi-centre)
NCT02006472**†**	PRIDE HD	Pridopidine	Sigma-1 receptor activation	Early and moderate HD	Placebo	Efficacy at 26 weeks	Randomized, double-blind, parallel assignment, dose-finding trial	408	Prilenia Therapeutics / Teva	Australia, Austria, Canada, Denmark, France, Germany, Italy, Poland, Russia, Netherlands, UK, USA (multi-centre)
NCT01306929**†**	OPEN-HART	Pridopidine	Sigma-1 receptor activation	HD	None	Safety up to 72 months	Randomized, placebo-controlled, dose-ranging, parallel-group study.	134	Prilenia Therapeutics / Teva	Canada, USA (multi-centre)
NCT05509153	NAC-preHD	NAC	Antioxidant	Premanifest HD	Placebo	Efficacy at 36 months	Randomized, double-blind trial	160	Western Sydney Local Health District	Australia (multi-centre)
ISRCTN56240656**†**	FELL-HD	Felodipine	Calcium channel blocker	Early HD	None	Safety at 62 weeks	Non-randomised, multiple dose trial	18	Cambridge University	UK (single centre)
NCT05358821**†**	SURVEYOR	SAGE-718	Positive allosteric modulator of NMDA	Early and moderate HD	Placebo	Change in cognition at 28 days	Double-blind, placebo-controlled, single dose design trial	69	Sage Therapeutics	USA (multi-centre)
NCT05358717	PIVOT HD	PTC518	Small molecule splicing modulator	Premanifest, prodromal and early HD	Placebo	Safety at 113 days	Randomized, double-blind, placebo controlled, parallel assignment, multiple dose trial	162	PTC Therapeutics	France, Germany, Netherlands, UK, USA (multi-centre)
NCT05475483	-	Bevantolol hydrochloride (SOM-3355)	Beta-blocker	Early and moderate HD	Placebo	Efficacy at 8 weeks	Randomized, double-blind, placebo-controlled, parallel assignment multiple-dose trial	129	SOM Biotech	France, Germany, Italy, Poland, Spain, Switzerland, UK (multi-centre)
ACTRN12621001755820	-	Trehalose (SLS-005)	Disaccharide	Early HD, ALS, SCA3	None	Efficacy at 24 weeks	Non-randomized, open-label	15-18 (4 ALS, 10 HD, 4 SCA3)	Seelos Therapeutics	Australia (two centres)
NCT05541627	-	AB-1001 (BV-101)	AAV encoding for CYP46A1, enzyme converting cholesterol to 24-OH-cholesterol	Early HD	None	Safety at week 52	Non-randomized, open-label, sequential, single ascending dose	18	AskBio/ BrainVectis	France (single centre)
NCT05107128**†**	DIMENSION	SAGE-718	Positive allosteric modulator of NMDA	Early and moderate HD	Placebo	Change in cognition at 84 days	Double-blind, placebo-controlled, single dose design	189	Sage Therapeutics	Australia, Canada, USA (multi-centre)
NCT05111249**†**	VIBRANT HD	Branaplam	Small molecule splicing modulator	Early HD	Placebo	Reduction of mHTT protein at week 17 Safety at 104 weeks	Double-blind, placebo-controlled multiple dose design	75	Novartis Pharmaceuticals	Belgium, Canada, France, Germany, Hungary, Italy, Spain, UK, USA (multi-centre)
NCT05032196**†**	SELECT-HD	WVE-003	Allele-selective ASO	Early HD	Placebo	Safety at 36 weeks	Randomized, double-blind, placebo-controlled, combined single ascending dose/multiple ascending dose trial	36	Wave Life Sciences Ltd	Australia, Canada, Denmark, France, Germany, Poland, Spain, UK (multi-centre)
NCT05243017	—	AMT-130	rAAV5-miHTT	Early HD	None	Safety at 6 months	Non-randomized, sequential ascending, multiple-dose trial	15	uniQure Biopharma B.V.	Germany, Poland, UK (multi-centre)
NCT04713982	—	Deutetrabenazine	VMAT2 inhibitor	HD with chorea	None	Change in speech outcome at 10 weeks	Single-arm open-label trial	30	Vanderbilt University Medical Center	USA (single centre)
NCT04826692	—	Metformin	Antihyperglycemic / AMPK activator	Early and moderate HD	Placebo	Change in cognition at 52 weeks	Randomized, parallel assignment, double-blinded trial	60	Instituto de Investigación Sanitaria La Fe	Spain (single centre)
NCT04514367**†**	—	ANX005	C1q inhibitor	Early HD	None	Safety at 36 weeks	Single-dose open-label trial	28	Annexon, Inc	USA (multi-centre)
NCT04421339**†**	—	Melatonin	Melatonin receptor agonist	HD with sleep disturbance	Placebo	Sleep quality at 9 weeks	Randomised, cross-over, single-blinded (participant/caregiver)	20	The University of Texas Health Science Center, Houston	USA (single centre)
NCT04400331	—	Valbenazine	VMAT2 inhibitor	Early and moderate HD	None	Safety at 104 weeks	Open-label, single arm trial	150	Neurocrine Biosciences	USA, Canada (multi-centre)
NCT04301726	—	Deutetrabenazine	VMAT2 inhibitor	HD with dysphagia	Placebo	Dysphagia at 18 months	Randomized, parallel assignment, triple blinded trial	48	Fundación Huntington Puerto Rico	N/S
NCT04478734; CTIS2023-508637-14-00	HUNTIAM	Thiamine and biotin	B vitamins	HD	Moderate vs high doses of thiamine and biotin	Safety at 52 weeks	Randomized, parallel assignment, open-label trial	24	Fundación Pública Andaluza para la gestión de la Investigación en Sevilla	Spain (single centre)
NCT04201834**†**	—	Risperidone	Dopamine antagonist	Early and moderate HD with chorea	None	Change in motor scales at 12 weeks	Non-randomized, open-label (assessor-blind), uncontrolled trial	12	University of Rochester	USA (single centre)
NCT04071639	—	Haloperidol, risperidone, sertraline and coenzyme Q10	Multiple (dopamine antagonists, selective serotonin reuptake inhibitor, dietary supplement)	Early and moderate HD	Coenzyme Q10	Efficacy at 5 years	Randomized, open-label, controlled, parallel trial	100	Second Affiliated Hospital, School of Medicine, Zhejiang University	China (single centre)
NCT04120493	AMT-130	rAAV5-miHTT	Non-allele-selective miRNA	Early HD	Sham intervention	Safety at 18 months	Randomized, double-blind, sham-controlled, parallel trial	26	uniQure Biopharma B.V.	USA (multi-centre)
NCT04102579**†**	KINECT-HD	Valbenazine	VMAT2 inhibitor	HD with chorea	Placebo	Efficacy at 12 weeks	Randomized, double-blind, placebo-controlled, parallel trial	120	Neurocrine Biosciences, Huntington Study Group	USA (multi-centre)
EUCTR2019-002178-30-DK**†**	—	WVE-120102	Allele-selective ASO	HD	None	Safety and tolerability at 97 weeks	Open-label extension	70	Wave Life Sciences Ltd	Australia, Canada, Denmark, France, Poland, UK (multi-centre)
NCT04000594**†**	GEN-PEAK	RG6042	Allele-nonselective ASO	HD	None	Pharmacodynamics and pharmacokinetics at multiple timepoints until 6 months	Non-randomized. open-label, multiple-dose, parallel trial	20	Hoffmann-La Roche	Netherlands, UK (multi-centre)
NCT03980938**†**	—	Neflamapimod	p38α MAPK inhibitor	Early HD	Placebo	Change in cognitive scales at 10 weeks	Randomized, double-blind, placebo-controlled, cross-over trial	16	EIP Pharma Inc, Voisin Consulting, Inc.	UK (single centre)
NCT03842969**†**	GEN-EXTEND	RG6042	Allele-nonselective ASO	HD	None	Safety and tolerability at up to 5 years	Open-label extension	1050	Hoffmann-La Roche	USA, Canada, Europe (multi-centre)
NCT03761849**†**	GENERATION HD1	RG6042	Allele-nonselective ASO	HD	Placebo	Clinical efficacy at 101 weeks	Randomized, double-blind, placebo-controlled, parallel trial	909	Hoffmann-La Roche	USA, Canada, Europe (multi-centre)
NCT03515213**†**	—	Fenofibrate	PPARα agonist	HD	Placebo	Pharmacodynamics at 6 months	Randomized, double-blind, placebo-controlled, parallel trial	20	University of California, Irvine	USA (single centre)
NCT03764215	Tasigna HD	Nilotinib	Selective Bcr-Abl tyrosine kinase inhibitor	HD	None	Safety, tolerability and pharmacodynamics at 3 months	Open-label, multiple ascending dose	20	Georgetown University	USA (single centre)
NCT03225833**†**	PRECISION-HD1	WVE-120101	Allele-selective ASO	HD	Placebo	Safety and tolerability at 1 and 120 days	Randomized, double-blind, placebo-controlled, combined single ascending dose/multiple ascending dose trial	48	Wave Life Sciences Ltd	Australia, Canada, Denmark, France, Poland, UK (multi-centre)
NCT03225846**†**	PRECISION-HD2	WVE-120102	Allele-selective ASO	HD	Placebo	Safety and tolerability at 1 and 120 days	Randomized, double-blind, placebo-controlled, combined single ascending dose/multiple ascending dose trial	60	Wave Life Sciences Ltd	Australia, Canada, Denmark, France, Poland, UK (multi-centre)
NCT02453061**†**	TRIHEP 3	Triheptanoin	Anaplerotic therapy	HD	Safflower oil	Pharmacodynamic efficacy at 6 months	Randomized, double-blind, controlled, parallel trial	100	Institut National de la Santé Et de la Recherche Médicale, Ultragenyx Pharmaceutical Inc	France, Netherlands (multi-centre)
NCT02509793	—	Tetrabenazine	VMAT2 inhibitor	HD with impulsivity	None	Cognitive and behavioural effects at 8 weeks	Single group, open-label trial	20	University of Texas Health Science Center, and H. Lundbeck A/S	USA (single centre)
NCT02481674**†**	SIGNAL	VX15/2503	Anti-semaphorin 4D monoclonal antibody	Late premanifest or early HD	Placebo	Safety and tolerability at 15 and 21 months	Randomized, double-blind, placebo-controlled, parallel trial	240	Vaccinex Inc., Huntington Study Group	USA (multi-centre)
EUCTR2013-002545-10-SE	OSU6162Open1309	(-)-OSU616	Monoaminergic stabilizer	HD, PD, brain trauma, stroke, myalgic encephalomyelitis and narcolepsy	None	Safety at 3, 6 and 12 months	Single group, open-label trial	240	A. Carlsson Research AB	Sweden (multi-centre)
NCT00514774	UDCA-HD	Ursodiol	Bile acid	HD	Placebo	Safety, tolerability, and pharmacokinetics at 35 days	Randomized, double-blind, placebo-controlled, parallel trial	21	Oregon Health and Science University, Huntington Study Group, Huntington Society of Canada	N/S

AAV: adeno-associated virus; AMP: adenosine monophosphate; AMPK: AMP-activated protein kinase; ASO: antisense oligonucleotide; HD: Huntington's disease; HD-ISS: Huntington's Disease Integrated Staging System; HTT: huntingtin; MAPK: mitogen-activated protein kinase; mHTT: mutant huntingtin; miRNA: microRNA; MRI: magnetic resonance imaging; mRNA: messenger ribonucleic acid; NAC: N-acetylcysteine; NAD^+^: nicotinamide adenine dinucleotide (oxidised form); NMDA: N-methyl-D-aspartate; N/S: not specified; PD: Parkinson's disease; PMS1: post-meiotic segregation 1; PPARα: peroxisome proliferator-activated receptor alpha; RNA: ribonucleic acid; SCA1: spinocerebellar ataxia 1; SCA3: spinocerebellar ataxia 3; TD: tardive dyskinesia; TEAEs: treatment-emergent adverse events; TFC: Total Functional Capacity; UHDRS: Unified Huntington's Disease Rating Scale; VMAT2: vesicular monoamine transporter 2. Note: IONIS-HTT_Rx_, ISIS 443139, RG6042 and tominersen refer to the same molecule. New trials added since the last Clinical Trials Update are indicated by *. Pharmacological trials terminated are indicated by †.

**Table 3.** Invasive non-pharmacological clinical trials registered at the WHO ICTRP for people with HD since the first edition of the “Huntington's Disease Clinical Trials Corner”.

Registration ID	Trial Name	Intervention	Mechanism of Action	Population	Comparison	Main Outcome	Study Design	Estimated Enrolment	Sponsor	Location(s)
NCT06444217	FibroTG-HD	Skin biopsy	Skin biopsy	Individuals with a CAG ≥36 allele (with reduced or full penetrance)	None	In vitro validation of a RNA trans-splicing gene therapy for the correction of supernumerary CAG repeats into fibroblasts derived from skin biopsies	Open-label, single group assignment	20	University Hospital, Angers	France (single centre)
NCT06097780	—	Nestacell	Dental pulp stem cell	Early and moderate HD	Placebo	Efficacy at 1 year	Randomized, double-blind, parallel assignment, multiple dose	120	Azidus Brasil	N/S
NCT04244513	—	GPi DBS	DBS	HD with chorea	Sham intervention	Efficacy at 3 and 6 months	Randomized, double-blind, sham-controlled, cross-over trial	40	Beijing Municipal Administration of Hospitals, Medtronic	China (multi-centre)
NCT04219241	ADORE-EXT	Cellavita	Stem cell therapy	HD	None	Efficacy and safety at 2 years	Open-label extension	35	Azidus Brasil, Cellavita Pesquisa Científica Ltda	Brazil (single centre)
ISRCTN52651778	TRIDENT	Foetal stem cell transplant	Stem cell therapy	Early HD	Usual care	Safety at 4 weeks	Randomized, open-label, controlled, parallel trial	30	Cardiff University, UK	UK (single centre)
NCT02728115	SAVE-DH	Cellavita	Stem cell therapy	HD	None	Safety at 5 years	Non-randomized, open-label, uncontrolled, parallel trial	6	Azidus Brasil	Brazil (single centre)
NCT03252535	ADORE-HD	Cellavita	Stem cell therapy	HD	Placebo	Efficacy at 120 days	Randomized, double-blind, placebo-controlled, parallel trial	35	Azidus Brasil	Brazil (single centre)
NCT03297177	—	Autologous stem/stromal cells	Autologous stem/stromal cell injection	HD, AD, PD, CBD, MS	None	Safety at 5 years	Single group, open-label trial	300	Healeon Medical Inc, Global Alliance for Regenerative Medicine, Regeneris Medical	Honduras, USA (multi-centre)
NCT02535884	HD-DBS	GP DBS	DBS	Moderate HD with chorea	Sham intervention	Efficacy at 12 months	Randomized, double-blind, sham-controlled, parallel trial	50	Heinrich-Heine University, KKS Netzwerk, Medtronic, The George Institute, EHDN, CHDI Foundation, Inc.	Austria, France Germany, Switzerland (multi-centre)
NCT01834053	BMACHC	Bone marrow derived MNC transplant	Bone marrow transplant	HD with chorea	None	Cognitive and behavioural effects at 6 months	Single group, open-label trial	50	Chaitanya Hospital, Pune	India (single centre)
NCT02252380	—	MR-guided focused ultrasound	Extracranial stereotactic radioablation	HD, ET, HT, PD, WD, dystonia, TD or orofacial dyskinesias	None	Adverse events after the procedure	Single group, open-label trial	10	InSightec	Canada (single centre)

AD: Alzheimer's disease; CBD: corticobasal degeneration; DBS: deep brain stimulation; ET: essential tremor; GP: globus pallidus; GPi: globus pallidus internus; HD: Huntington's disease; HT: Holmes tremor; ICTRP: International Clinical Trials Registry Platform; MNC: mononuclear cells; MR: magnetic resonance; MS: multiple sclerosis; N/S: not specified; RNA: ribonucleic acid; PD: Parkinson's disease; TD: tardive dyskinesia; WD: Wilson's disease; WHO: World Health Organization.

**Table 4.** Non-invasive non-pharmacological clinical trials registered at the WHO ICTRP for people with HD since the first edition of the “Huntington's Disease Clinical Trials Corner”.

Registration ID	Trial Name	Intervention	Mechanism of Action	Population	Comparison	Main Outcome	Study Design	Estimated Enrolment	Sponsor	Location(s)
NCT07136844*	Acti-Adult	Syde^®^ wearable device; standardised functional tests; disease-specific clinical scales	Digital mobility and activity monitoring with magneto-inertial sensors to capture real-world gait and upper limb function	Ambulatory adults with neurological or metabolic movement disorders (including HD) and obesity	Parallel disease cohorts	95^th^ centile of stride velocity (SV95C) every 6 months for up to 2 years	Non-randomized, parallel assignment, open-label; diagnostic purpose	300	Centre Hospitalier Universitaire de Liège	Belgium (single centre)
NCT07010705*	MEND-HD	ActiGraph LEAP (wearable device); Axivity AX6 (wearable device)	Digital health technologies to quantify gait, mobility and chorea (wearable sensor-based endpoints)	HD-ISS stage 2 or early stage 3	Healthy controls	Known-group validity and inter-method reliability of digital measures of stride time variability and total time in chorea	Observational (case-control, prospective)	100	University of Rochester	USA (single centre)
NCT06843252*	—	Active tDCS	Non-invasive brain stimulation modulating cortical excitability and frontostriatal networks to improve cognition and behaviour	Early and moderate HD	Sham tDCS (crossover)	Study completion, acceptability, side effects; secondary: cognition (FAB), behaviour (PBA-s)	Randomized, double-blind, sham-controlled, crossover pilot trial	16	The University of Texas Health Science Center at San Antonio	USA (single centre)
NCT06807892*	CARE-MH	Synergistic management (simultaneous cognitive-motor exergames); sequential management (separate cognitive then motor training)	Cognitive-motor training via exergames aiming to improve neuroplasticity, balance, motor and cognitive function	Early HD	Synergistic vs sequential management	Berg Balance Scale (BBS)	Randomized, parallel-group, open-label supportive care trial	40	University Hospital, Angers	France (single centre)
NCT06774443	HT-HD	Neuropsychological assessment (Hinting Task)	Assessment of social cognition deficits, specifically Theory of Mind	HD	Healthy controls	Sensitivity of the Hinting Task in detecting social cognition impairments in HD	Observational (case-control, cross-sectional)	52	University Medical Center Groningen	Netherlands (single centre)
NCT06693466	HD-P1	Experimental pain assessment (pain facilitation, facial expression, and conditioned pain modulation)	Assessment of pain processing mechanisms and sensory modulation in HD	HD	None	Feasibility of experimental pain assessment protocols in HD	Observational (cohort, cross-sectional)	20	Leiden University Medical Center	Netherlands (single centre)
NCT06634628	iMagemHTT-009	PET imaging with novel radioligand [^11^C]CHDI-00491009	Radioligand binding to aggregated mHTT for quantification	HD	None	VT of [^11^C]CHDI-00491009 measured via PET imaging	Non-randomized, sequential assignment	27	CHDI Foundation, Inc.	Belgium (single centre)
NCT06626308	BIO-MH	Brain fMRI and gait assessment using 3D virtual reality	fMRI to assess early subcortical biomarkers and virtual reality-based gait analysis for detecting preclinical motor abnormalities	Premanifest HD	Healthy controls	BOLD signal changes in subcortical regions and alterations in gait parameters	Observational (case-control)	20	University Hospital, Grenoble	France (single centre)
NCT06626412	—	PET of SV2A using the radioligand 18F-SynVesT-1	Radioligand binding to SV2A for quantification of synaptic density	Late premanifest HD	Volumetric MRI	Baseline differences and longitudinal changes in synaptic density and brain volume	Non-randomized, single-group assignment	35	Universitaire Ziekenhuizen KU Leuven	Belgium (single centre)
NCT06585332	—	Hand grip strength as a screening tool for respiratory dysfunction	Assessment of neuromuscular function related to respiratory effort	HD	None	Maximal hand grip strength, voluntary peak cough flow, maximal expiratory pressure, maximal inspiratory pressure	Observational (case only)	70	General University Hospital	Prague (single centre)
NCT06546488	CAT-HD	Behavioral assessments	Standardised assessment batteries using the DSCT and SAGE	HD	SDMT, SWR	SDMT compared to DSCT; SAGE score compared to SDMT and SWR scores	Observational (case only)	76	Ohio State University	USA (single centre)
NCT06490367	TREHD	TRE diet	TRE diet, specifically maintaining a 6-8-h eating window every day for 12 weeks	Premanifest, prodromal and early HD	None	Change from baseline in the daily eating period at week 13	Prospective interventional, open-label, single-arm trial	25	Oregon Health and Science University	USA (single centre)
NCT06414967	MUSIC-HD	Music therapy	Music therapy using a digital music therapy tool combined with conventional management	Early HD	None	Change in irritability after 3 months	Single group, open-label	15	Poitiers University Hospital	France (single centre)
RBR-75ys4s9	—	Dance therapy	Dance sessions offered by physiotherapists using auditory and visual stimuli	HD, PD, dystonia	Standard medical treatment	Quality of life improvement	Two-arm randomized controlled trial with a blinded examiner	100	Universidade Federal de Juiz de Fora	Brazil (single centre)
ChiCTR2300069844	—	Repetitive transcranial magnetic stimulation	Transcranial magnetic stimulation	HD	None	EEG	Non-randomized, open-label, single group trial	20	Shenzhen People's Hospital	China (single centre)
ISRCTN47330596	—	Psychological intervention	Guided self help	Premanifest and manifest HD	Usual treatment	Feasibility at 3 and 6 months	Interventional randomized controlled trial	30	Leicestershire Partnership NHS Trust, UK	UK (single centre)
RBR-463yhb3	—	Multimodal physiotherapy	Balance intervention with rhythmic cues	HD	Educational program	Balance	Randomized, double-blinded, parallel assignment trial	36	São Paulo University, Brazil	Brazil (single centre)
ACTRN12622000908730	—	Online platform	Computerized cognitive training	Premanifest and early HD	Lifestyle education	Change in cognition at 12 weeks	Randomized, blinded (investigator, statistician) parallel assignment trial	50	Monash University, Australia	Australia (two centres)
ISRCTN11906973	HD-DRUM	Training app	Drumming	Premanifest, early and moderate HD	Standard medical care	Feasibility	Randomized, parallel assignment trial	50	Cardiff University, UK	UK (single centre)
NCT05326451	—	Transcranial direct current stimulation	Transcranial electrical stimulation	Early and moderate HD	None	Treatment completion, acceptability and safety	Non-randomized, open-label, single group trial	10	The University of Texas Health Science Center, Houston, USA	USA (single centre)
ACTRN12622000345785	—	Multidisciplinary therapy coaching program	Education	Premanifest and early HD	Lifestyle guidance	Barriers and motivators to engagement in telehealth interventions and digital health literacy	Randomized, single blind, parallel assignment trial	84	Perpetual Limited	Australia (two centres)
NCT04917133	HUNT'ACTIV	Adapted physical workshops plus classic 4-week rehabilitation program	Physical activity, cycling, horse riding, situation tests, cultural outings	Mid-stage HD	Classic 4-week rehabilitation program	Motor function at 1 month	Randomized, parallel assignment trial	32	Assistance Publique - Hôpitaux de Paris	France (single centre)
NCT04429230	—	Transcranial pulsed current stimulation	Transcranial electrical stimulation	HD	Sham intervention	Feasibility at one year	Randomized, crossover double-blinded trial	15	Western University, Canada	N/S
ACTRN12620000281998	—	Ketogenic diet	Ketogenic diet	HD	None	Change in cognition and motor scores at 12 weeks	Non-randomized, open-label, single group trial	10	Waikato Hospital	New Zealand
ACTRN12619000870156	—	Transcranial alternating current stimulation	Transcranial magnetic stimulation	Premanifest and early HD	Sham intervention	Biomarkers	Randomized, open-label, cross-over trials	60	Monash University, Epworth Centre for Innovation in Mental Health	Australia (single centre)
ACTRN12618001717246	—	Multidisciplinary therapy program	Exercise, cognitive training, lifestyle guidance and social activities	Premanifest HD	Standard of care	Feasibility and safety	Clustered, non-randomized, open-label, parallel trial	40	Edith Cowan University, Deakin University and Lotterywest	Australia (two centres)
NCT03417583	—	Neuropsychiatric treatment protocol	Multidisciplinary intervention	HD with neuropsychiatric symptoms	Standard of care	Change in quality of life at 18 months	Non-randomized, assessor-blinded, parallel trial	100	Vanderbilt University Medical Center and Teva Pharmaceuticals USA	USA (single centre)
CTRI/2018/01/011359	—	Repetitive transcranial magnetic stimulation	Transcranial magnetic stimulation	Early to moderate HD and PD	Sham stimulation	Efficacy at 5 days	Randomized, single-blind, placebo-controlled, parallel trial	40	Vinay Goyal	India (single centre)
NCT03344601	PACE-HD	Supported structured aerobic exercise training program	Physiotherapy	HD	Activity as usual	Data completeness, recruitment, retention, safety, adherence, fidelity and acceptability at 12 months	Nested open-label, randomized controlled parallel trial	120	Cardiff University and CHDI Foundation, Inc	Germany, Spain, USA (multi-centre)
ACTRN12617001269325	—	Swallowing skill training	Speech and language therapy	HD and ALS	None	Swallowing function and quality of life at 2 weeks	Single group, open-label trial	54	University of Canterbury	New Zealand (single centre)

3D: three-dimensional; AD: Alzheimer's disease; ALS: amyotrophic lateral sclerosis; BOLD: blood oxygen level-dependent; DSCT: Digit Symbol Coding Test; EEG: electroencephalography; ET: essential tremor; FAB: Frontal Assessment Battery; fMRI: functional magnetic resonance imaging; HD: Huntington's disease; HT: Holmes tremor; ICTRP: International Clinical Trials Registry Platform; mHTT: mutant huntingtin; MRI: magnetic resonance imaging; MS: multiple sclerosis; N/S: not specified; PBA-s: Problem Behaviors Assessment - short; PD: Parkinson's disease; PET: positron emission tomography; SAGE: Self-Administered Gerocognitive Examination; SDMT: Symbol Digit Modalities Test; SV2A: synaptic vesicle glycoprotein 2A; SWR: Stroop Word Reading; TD: tardive dyskinesia; tDCS: transcranial direct current stimulation; TRE: time-restricted eating; VT: volume of distribution; WHO: World Health Organization. New trials since the last Clinical Trials Update are indicated by *.

If you would like to draw attention to specific trials, please feel free to email us at: mena.farag@ucl.ac.uk and e.wild@ucl.ac.uk.

## Ongoing clinical trials

A list of all registered clinical trials is given in Tables 2–4.

### SKY-0515 (NCT06873334)^13^

**Study Title:** Study of SKY-0515 for Safety, Efficacy and Pharmacodynamics in Participants with Huntington's Disease (FALCON-HD).

**Intervention:** Oral SKY-0515 (low, mid, or high dose) or matched placebo, administered once daily for 12 months. SKY-0515 is an mRNA-splicing modulator designed to reduce both wild-type HTT and mutant huntingtin (mHTT) protein by lowering *HTT* mRNA levels, and to suppress post-meiotic segregation 1 (PMS1) protein, a mediator of somatic CAG repeat expansion and known driver of HD progression.^13,21,22^

**Description:** The FALCON-HD study, sponsored by Skyhawk Therapeutics, Inc., is a phase II/III randomised, double blind, placebo-controlled, dose ranging clinical trial to evaluate the pharmacodynamics, safety and efficacy of SKY-0515 in people with HD. This multi-centre clinical trial will enrol 120 adults (≥25 years) with genetically confirmed HD (CAG repeat length ≥40) who meet defined functional and motor thresholds (specifically Unified Huntington's Disease Rating Scale (UHDRS) Total Functional Capacity (TFC) ≥ 10; Total Motor Score (TMS) ≥ 6). The clinical trial design comprises a screening period lasting up to four weeks, a 12-month double-blind treatment period and a four-week follow-up period thereafter. Participants will be randomised (1:1:1:1) to one of three once-daily dose levels of SKY-0515 or placebo. Safety and efficacy will be assessed through regular clinical trial visits, laboratory tests, brain MRI and standardised clinical assessments, including the UHDRS.

Primary outcome measures are change from baseline to 12 months in blood mHTT protein levels, MRI-derived brain volumes and UHDRS scores. Other outcome measures include changes in other HD-related biomarkers and long-term safety of SKY-0515.

**Sponsor/Funders:** Skyhawk Therapeutics, Inc.

**EHDN Endorsed Trial:** No

**Comments:** On 9^th^ July 2024, Skyhawk Therapeutics announced positive results from its phase I trial of SKY-0515 (ACTRN12623001161617)^21^ in healthy volunteers, initiated in late 2023.^22^ SKY-0515 produced dose-dependent reductions in *HTT* mRNA, with maximum decreases of 67% after a single 16 mg dose and 72% after 14 days of 9 mg daily dosing.^22^ Reductions were observed across all dose levels, and the drug was generally well-tolerated. On 17^th^ September 2025, Skyhawk Therapeutics reported further positive findings from the first interim analysis of this phase 1 trial.^23^ At day 84, treatment with SKY-0515 resulted in dose-dependent reductions of mHTT protein level in blood, including a 29% reduction at the 3 mg dose and a 62% reduction at the 9 mg dose. SKY-0515 was generally well tolerated at both dose levels.^23^

On 20^th^ June 2025, Skyhawk Therapeutics announced that the first patient was dosed in the phase II/III FALCON-HD study (NCT06873334)^13^ in Adelaide, Australia,^24^ marking the initiation of the registrational trial programme across multiple sites in Australia and New Zealand.

SKY-0515 is designed to reduce both HTT and PMS1 proteins, the latter implicated in somatic CAG repeat expansion, a mechanistic process increasingly recognised as a key driver of neuronal loss in HD.^25,26^ Genome-wide association and mismatch repair studies indicate that targeting PMS1 and other DNA repair genes could help prevent progressive neuronal pathogenesis in striatal and cortical regions.^27,28^ This important phase II/III clinical trial of SKY-0515 will assess whether HTT and PMS1 lowering can offer greater therapeutic benefit than HTT reduction alone in early-stage HD.

### SPK-10001 (NCT06826612)^14^

**Study Title:** A Randomized Study of SPK-10001 Gene Therapy in Participants with Huntington's Disease.

**Intervention:** One-time bilateral intraparenchymal infusion of SPK-10001 (also known as RG6662) into the caudate and putamen, compared with placebo surgery.^14^ SPK-10001 is an engineered adeno-associated virus (AAV) vector delivering an artificial microRNA designed to selectively target human *HTT* mRNA.^29^

**Description:** The SPK-10001 clinical trial, sponsored by Spark Therapeutics, Inc., is a phase I/II multicentre, randomized, sequential, dose-escalation study evaluating the safety, tolerability, and preliminary efficacy of SPK-10001, an investigational gene therapy, in people with HD. Eligible participants receive a one-time bilateral intraparenchymal infusion of SPK-10001 into the caudate and putamen, or undergo a placebo surgical procedure. The clinical trial is structured in four sequential parts: an initial open-label cohort (Part A) in which all participants receive SPK-10001; a randomized, double-blind, placebo-surgery-controlled cohort (Part B); an open-label crossover (Part C) in which participants initially assigned to placebo surgery receive SPK-10001; and a long-term follow-up phase (Part D) for all participants following completion of active treatment. Up to 53 adults (aged 25–65 years) with genetically confirmed HD (CAG repeat length ≥40), evidence of striatal atrophy and preserved functional capacity (defined as UHDRS TFC ≥11) will be enrolled into the clinical trial.

Primary outcome measures are safety and tolerability, measured by the incidence and severity of treatment-emergent adverse events (TEAEs) over a five-year follow-up period, and change from baseline in UHDRS TFC score at 24 months. Secondary outcome measures include changes from baseline in motor progression assessed by digital motor testing at 12, 18 and 24 months; changes in composite UHDRS (cUHDRS) scores at 12, 18 and 24 months; and changes in UHDRS TFC scores at 12 and 18 months.

**Sponsor/Funders:** Spark Therapeutics, Inc.

**EHDN Endorsed Trial:** No

**Comments:** Data on SPK-10001 were presented at the 31^st^ Annual Meeting of the Huntington Study Group (HSG), held in Cincinnati, Ohio from 7^th^ to 9^th^ November 2024, and at the Cure Huntington's Disease Initiative (CHDI) 20^th^ Annual HD Therapeutics Conference, held in Palm Springs, California from 24^th^ to 27^th^ February 2025.^29,30^ Preclinical studies of SPK-10001, an engineered AAV vector delivering an artificial microRNA targeting human *HTT* mRNA, demonstrated a consistent, dose-dependent reduction of *HTT* mRNA and protein in HD mouse models following direct intrastriatal injection, compared with untreated controls. Reductions of up to 40% in *HTT* mRNA and up to 70% in *HTT* protein were well-tolerated and remained stable over a 12-month period. In contrast to uniQure's AMT-130 gene therapy, SPK-10001 targets an RNA sequence downstream of exon 1, promising valuable insights into the relative importance of the exon 1a splice variant as opposed to full length mHTT. In nonhuman primate studies, robust and consistent suppression of *HTT* protein throughout the caudate and putamen was achieved only through direct intraparenchymal administration, whereas intra cerebrospinal fluid (CSF) routes (intraventricular, intrathecal or intracisternal) failed to achieve satisfactory target engagement in these regions. Safety data in cynomolgus macaques indicated that the surgical procedure was generally well-tolerated with minimal perioperative immunosuppression. Transient elevations in CSF neurofilament light chain (NfL) protein were observed in all animals but returned to baseline within 12 months. Levels of *HTT* suppression were stable over the same period and highly consistent within dose groups.^29^ On 12^th^ June 2025, Spark Therapeutics and Roche issued a joint community update via a letter announcing that the first participant had been dosed with RG6662, also known as SPK-10001, and that the programme will be fully transitioned to Roche.^31^

These findings and the update from Spark Therapeutics and Roche represent an important step in the development and progress of gene therapy for HD, particularly in light of the news below from the first gene therapy to be tested in HD patients. Early preclinical data support target engagement and tolerability of SPK-10001; however, safety, efficacy and clinical benefit in humans remain to be established. Long-term monitoring will be essential to determine the durability of any therapeutic effects, ongoing safety and potential functional impact as results from the ongoing phase I/II clinical trial become available.

## Breaking news

In this section we provide brief updates about ongoing or recently terminated clinical trials.

The **AMT-130** (NCT04120493; NCT05243017)^15,16^ study, sponsored by uniQure Biopharma B.V., is investigating the effects of AMT-130, a one-time intracranially administered gene therapy that uses an AAV vector (AAV5-miHTT) to deliver a microRNA targeting the *HTT* gene. The treatment is intended to provide long-lasting supression of HTT expression. On 24^th^ September 2025, uniQure released positive topline results from the phase I/II clinical trial of AMT-130.^32^ The prespecified analysis included 29 treated participants (17 high-dose, 12 low-dose), with 12 patients per cohort reaching 36 months of follow-up. Outcomes were compared with propensity score-matched external controls from the Enroll-HD natural history dataset (n = 940 for high dose; n = 626 for low dose). At 36 months, high-dose AMT-130 met the primary endpoint, demonstrating a statistically significant 75% slowing of disease progression versus controls, as measured by the cUHDRS (*P* = 0.003; mean change from baseline −0.38 vs −1.52). A key secondary endpoint, UHDRS TFC, showed a 60% slowing of decline (*P* = 0.033; −0.36 vs −0.88). Additional secondary measures indicated favourable trends, including Symbol Digit Modalities Test (SDMT) (88% slowing; *P* = 0.057; −0.44 vs. −3.73), Stroop Word Reading (SWR) (113% slowing; *P* = 0.002; 0.88 vs. −6.98) and UHDRS TMS (59% slowing; *P* = 0.174; 2.01 vs. 4.88). Fluid biomarker analysis also showed a mean reduction of 8.2% in CSF NfL from baseline at 36 months, which provides supportive biomarker evidence of target engagement and disease modification. AMT-130 was overall well-tolerated, with an acceptable safety profile observed in both the high-dose and low-dose cohorts.^32^ The sponsor reported that the consistently sustained improvements across functional, motor and cognitive outcomes at 36 months in the high-dose cohort, compared with the more variable findings at the lower dose, suggest a dose-dependent effect of AMT-130. Overall, the press release indicates that AMT-130 may have disease-modifying potential in HD and has the potential to meaningfully slow disease progression. The sponsor has also provisionally announced plans to submit a Biologics Licence Application to the US Food and Drug Administration in early 2026, with a potential US launch later that year pending regulatory approval.^32^ Peer-reviewed publication is expected and will permit close scrutiny of all the data, but the topline results appear to be the most encouraging seen so far in a clinical trial of a therapy intended to deflect the course of HD.

The **GENERATION HD2** (NCT05686551)^17^ study, sponsored by Roche, released an update on 17^th^ April 2025 regarding the tominersen programme.^33^ Tominersen is an intrathecally administered antisense oligonucleotide (ASO) therapy designed to lower *HTT* expression. GENERATION HD2 is a phase II, randomised, double-blind, placebo-controlled, dose-finding clinical trial comparing 60 mg tominersen, 100 mg tominersen and placebo. In the most recent update provided by Roche, the Independent Data Monitoring Committee (IDMC) reported that tominersen continues to demonstrate an acceptable safety profile and recommended continuation of the trial, while advising discontinuation of the 60 mg arm while continuing with the 100 mg dose, which was judged more likely to provide clinical benefit.^33^ This update is encouraging following the IDMC decision in March 2021 to halt the phase III GENERATION HD1 (NCT03761849)^34^ study, due to an unfavourable benefit-risk profile.^35^ In GENERATION HD2 (NCT05686551),^17^ participants who had been receiving the 60 mg dose will transition to the 100 mg dose in a blinded manner, while those in the placebo group will remain in their original assignment. Although this update does not establish efficacy, it represents a data-driven refinement of the study design to prioritise the dose most likely to demonstrate therapeutic benefit. The clinical trial remains ongoing, and definitive conclusions regarding clinical benefit will only be possible once the data are unblinded and fully analysed. This is anticipated to be late 2026.

The **PIVOT-HD** (NCT05358717; NCT06254482)^18,19^ study, sponsored by PTC Therapeutics, released an update on 5^th^ May 2025 regarding PTC518 (also known as votoplam), an orally administered huntingtin mRNA splice modulator.^36^ The study met its primary endpoint, showing dose-dependent reductions in blood HTT protein levels at week 12, along with a favourable safety and tolerability profile across all dose levels and disease stages. Results from the full 12-month cohort show that HD-ISS stage 2 participants showed dose-dependent reductions in blood *HTT* protein (up to 39% with the 10 mg dose) and emerging trends suggesting potential clinical benefit, including favourable changes on the composite UHDRS and TMS subscale. For HD-ISS stage 3 participants, more modest trends were observed, with apparent benefit in the 5 mg group but less consistent effects in the 10 mg group, raising the possibility that treatment response may differ by disease stage. At 24 months, data from an early cohort (n = 21) demonstrated dose-dependent reductions in plasma NfL levels (−8.9% at 5 mg and −14% at 10 mg) and favourable trends on cUHDRS, TFC and SDMT subscales, compared with a propensity-matched natural history cohort from the Enroll-HD registry. Importantly, for all dose levels and disease stages, PTC518 showed a favourable safety and tolerability profile with no treatment-related serious adverse events or NfL spikes observed. These findings support ongoing investigation of PTC518, which is currently being assessed in the open-label extension (NCT06254482).^19^ While these results are promising and suggestive of early signs of clinical benefit, definitive evidence of efficacy, functional impact and long-term safety will require confirmation in the ongoing open-label extension and potential subsequent phase III study. On that note, Novartis released a community update on 15^th^ July 2025 announcing a global licence and collaboration agreement with PTC Therapeutics for the development of votoplam (PTC518).^37^ Under this agreement, Novartis will assume sponsorship of the long-term extension study and lead any future phase III development. Novartis has indicated that analysis of the phase II data is ongoing and that discussions with regulatory authorities, clinical experts and patient advocacy groups are underway to inform the design of the next phase of development.

On 25^th^ July 2025, Prilenia and Ferrer announced that the European Medicines Agency's Committee for Medicinal Products for Human Use had recommended refusal of marketing authorisation for pridopidine in HD.^38^ This decision follows the outcome of the phase III **PROOF-HD** (NCT04556656)^20^ study, which enrolled 499 participants and evaluated a single daily dose of 45 mg pridopidine versus placebo. The primary endpoint, change in UHDRS TFC score from baseline to week 65, was not met, indicating no significant slowing of disease progression over the 15-month treatment period.^38^
